# Development and Systematic Evaluation of a Progressive Web Application for Women With Cardiac Pain: Usability Study

**DOI:** 10.2196/57583

**Published:** 2025-04-17

**Authors:** Monica Parry, Tony Huang, Hance Clarke, Ann Kristin Bjørnnes, Paula Harvey, Laura Parente, Colleen Norris, Louise Pilote, Jennifer Price, Jennifer N Stinson, Arland O’Hara, Madusha Fernando, Judy Watt-Watson, Nicole Nickerson, Vincenza Spiteri DeBonis, Donna Hart, Christine Faubert

**Affiliations:** 1 Lawrence Bloomberg Faculty of Nursing University of Toronto Toronto, ON Canada; 2 Pain Research Unit University Health Network Toronto, ON Canada; 3 University of Toronto Toronto, ON Canada; 4 Department of Nursing and Health Promotion Oslo Metropolitan University Oslo Norway; 5 Women’s College Hospital Toronto, ON Canada; 6 Healthcare Human Factors University Health Network Toronto, ON Canada; 7 Faculty of Nursing University of Alberta Edmonton, AB Canada; 8 Department of Medicine McGill University Research Institute of the McGill University Health Centre Montreal, QC Canada; 9 Research Institute The Hospital for Sick Children Toronto, ON Canada; 10 Patient Advisor LaHave, NS Canada; 11 Patient Advisor Toronto, ON Canada

**Keywords:** digital health, chatbot, women, cardiac pain, usability testing, self-management, artificial intelligence, AI

## Abstract

**Background:**

Cardiac pain has been widely considered to be the primary indicator of coronary artery disease. The presentation of cardiac pain and associated symptoms vary in women, making it challenging to interpret as cardiac, possibly cardiac, or noncardiac. Women prefer to consult with family and friends instead of seeking immediate medical care.

**Objective:**

This study aimed to assess the user performance (ie, ease of use, efficiency, and errors) and user satisfaction (System Usability Scale; SUS) of a progressive web application for women with cardiac pain.

**Methods:**

Following ethics approval, a purposive sample of women aged >18 years with cardiac pain or associated symptoms lasting >3 months and able to speak and read English was recruited to participate in 2 iterative usability testing cycles. The first cycle assessed the performance of and satisfaction with *at heart* using a web application, and the second cycle assessed the performance of and satisfaction with *at heart* across various Android and iOS devices. In total, 2 investigators recorded user comments and documented problems. At the end of the testing session, the participants completed the SUS and 4 semistructured interview questions.

**Results:**

In total, 10 eligible women participated in usability testing from March 31, 2020, to April 17, 2020 (cycle 1), and from November 17, 2020, to November 30, 2020 (cycle 2). Women across usability testing cycles had a mean age of 55.6 (SD 7.3) years, and most (9/10, 90%) were well educated. In total, 50% (5/10) were employed full or part time, and 60% (6/10) earned >CAD $70,000 (US $48,881.80) annually. Participants across 2 testing cycles reported the overall usability of the *at heart* progressive web application as highly acceptable (mean SUS score 81.75, SD 10.41). In total, 90% (9/10) of participants rated the user-friendliness of *at heart* as good or excellent. All participants (10/10, 100%) thought *at heart* was easy to use and efficient. Only 2 testing errors were noted as high priority; these were low contrast or small font and clarification that the chatbot was not a real person. User satisfaction was assessed using themes that emerged from the debrief and 4 semistructured interview questions; *at heart* was engaging, comprehensive, understandable, credible, relevant, affirming, personalized, and innovative.

**Conclusions:**

This study provides initial support for the *at heart* progressive web application for women living with cardiac pain and symptoms. Ongoing evaluations in phases 3 and 4 should aim to examine the feasibility and acceptability of and the extent of engagement with the *at heart* core feature set: Heart Check, Wellness Check, and the library. In addition to assessing effectiveness in the phase-4 effectiveness-implementation hybrid trial (type I), describing and better understanding the context for implementation (eg, race and ethnicity and geography) will be necessary.

**International Registered Report Identifier (IRRID):**

RR2-10.1136/bmjopen-2019-033092

## Introduction

### Background

Cardiovascular diseases constitute the leading cause of mortality worldwide, exerting a substantial economic burden on the health care system [1-4]. As the most prevalent form of cardiovascular disease, coronary artery disease (CAD) is estimated to be associated with 8.93 million deaths annually worldwide across all ages [1]. CAD is a complex condition that varies in clinical presentation across sexes, with both obstructive (macrovascular) and nonobstructive (microvascular) CAD being associated with cardiac pain and other symptoms [5]. Although cardiac pain has been widely considered to be the primary indicator of CAD [6], cardiac pain and associated symptoms reported by women with CAD differ markedly from those reported by men [7,8]. The presentation of cardiac pain and associated symptoms may vary in frequency, pattern, and distribution in women [8], making it challenging to interpret as cardiac specific [9]. Similarly, women that undergo a percutaneous coronary intervention or cardiac surgery report higher prevalence of persistent pain of moderate to severe intensity after treatment as compared to men [10-12]. This cardiac pain is often described by women as sharp and burning and may present with varying extent of dyspnea, fatigue, anxiety, and discomfort that may radiate to the jaws, shoulders, back, and arms [6,8,13]. The manifestations of cardiac pain and associated symptoms contribute to substantial morbidity and impairments in health-related quality of life (HRQoL) in women [14,15].

The complex presentation of CAD in women poses a challenge for timely recognition and management of symptoms. Recent data have shown that women often delay seeking medical care when experiencing acute cardiac pain or associated symptoms, with time between symptom onset and emergency department arrival being 85 to 320 minutes [16]. Women report experiencing difficulties in interpreting, understanding, and attributing their cardiac pain or symptoms to CAD, preferring to consult with family and friends instead of seeking immediate medical care [17]. Moreover, women often hesitate to seek medical care for cardiac pain or associated symptoms as a result of their gendered roles as caregivers [17,18]. Women describe having gendered roles that they are unable to delegate, such as providing care for dependent family members [17,18]. The cumulative effect of symptom underrecognition and hesitancy in women leads to delayed care-seeking behaviors and an increased risk of major adverse cardiac events or mortality as compared to men [6]. As such, it is imperative to promote proper recognition, assessment, and management of symptoms in women to improve health outcomes and HRQoL.

Self-management programs are designed to engage users as active participants in the management of their conditions; they are key predictors of successful behavior change [19,20]. These interventions generally use educational strategies designed to assist users in achieving optimal knowledge, understanding beliefs, and skills, as well as providing meaningful social supports [21]. Digital health–based self-management programs have been developed and effectively used to help women manage weight [22-25], increase physical activity [26], monitor for perinatal depression, and assist with postpartum smoking cessation [27]. Many women describe digital health interventions as being novel and supportive [22] and effective in motivating healthy behaviors, reducing symptoms [28], and improving HRQoL [23]. However, there is a lack of evidence-informed digital health self-management programs specifically for women with CAD living with cardiac pain and associated symptoms [7], demonstrating a clear need for a digital self-management program.

**Figure 1:**
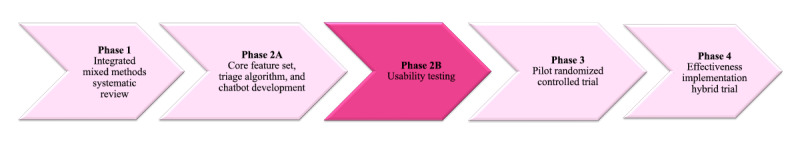
Phases of the *at heart* progressive web application development.

*At heart* (formerly HEARTPA♀N) [29], a self-management progressive web application, was developed for women with CAD using a sequential phased approach recommended by the Medical Research Council (MRC) [30-32]. In phase 1, an integrated mixed methods systematic review was conducted to evaluate the current evidence related to the self-management of cardiac pain and associated symptoms (eg, dyspnea and fatigue) in women [7,29]. The results of the review suggested that self-management interventions could reduce cardiac pain and associated symptoms if they targeted a greater proportion of women (standardized mean difference [SMD]=−0.01; SE 0.003; *P*=.02), goal setting (SMD=−0.26, 95% CI −0.49 to −0.03), and collaboration or support from a health care provider (SMD=−0.57, 95% CI −1.00 to −0.14) [33]. The review also identified a lack of self-management interventions targeted specifically for cardiac pain and associated symptoms in women [33]. In phase 2A, the content and core feature set, chatbot, and symptom triage algorithms were co-designed with health care professionals and women with CAD [29]. In phase 2B, the usability of *at heart* was evaluated to ensure that the platform was intuitive and acceptable for women with cardiac pain, and it is the focus of this paper. In phase 3, a process and preliminary efficacy evaluation using a 2-group parallel pilot randomized controlled trial (RCT) will be undertaken, and then a phase-4 effectiveness-implementation hybrid trial is planned (Figure 1).

Usability testing is an important phase in the development of digital health interventions [34,35]. End users test a prototype in iterative cycles; they provide feedback about what works, what does not work, and where gaps might exist in the information and functionality [34,35]. These factors contribute to the frequency of use, understanding, and acceptability and enhance the likelihood that users will use the end product [35,36]. Testing the usability of the intervention also serves to assess the suitability of the platform interface and content [37].

### Objectives

The objectives of this study were to assess the user performance (ie, ease of use, efficiency, and errors) and user satisfaction (interviews and System Usability Scale [SUS]) of a progressive web application for women with cardiac pain.

## Methods

### Participant Selection

Following ethics approval, a purposive sample of women was recruited from (1) an ambulatory care hospital focused on women’s health, (2) an adult tertiary care transitional pain clinic; (3) the Alberta Provincial Project for Outcome Assessment in Coronary Heart Disease registry; (4) the CorHealth Ontario Cardiac Registry; (5) established strategies through the Canadian Pain Coalition; (6) the Ontario Women’s Health Network’s listserve, which reaches >1900 women and community organizations in Ontario, Canada; and (7) social media. The Ontario Women’s Health Network regularly brought women who lived in rural and remote areas together, including women who were disabled, women of color, women with a low income, and Indigenous and older women. Women were eligible to participate in the usability testing if they (1) were aged >18 years, (2) had been diagnosed with obstructive or nonobstructive CAD pain or pain after a percutaneous coronary intervention or cardiac surgery lasting >3 months, (3) were able to speak and read English, and (4) had not previously used or tested the *at heart* progressive web application. Women were excluded from the study if they had (1) severe cognitive impairment, assessed using the Six-Item Screener administered via telephone; or (2) a major comorbid medical or psychiatric condition that would preclude their ability to participate in the usability testing. The Six-Item Screener is a brief instrument for identifying individuals with cognitive impairment, and its diagnostic properties are similar to those of the Mini-Mental State Examination [38,39].

### Ethical Considerations

The phase-2B usability testing was approved by the Health Sciences Research Ethics Board (REB; 36415) at the University of Toronto on November 26, 2018, and the Health Sciences and Affiliated Teaching Hospitals REB (6026830) at Queen’s University on June 20, 2019. An amendment was granted by the Health Sciences REB (36415) at the University of Toronto on March 26, 2020, to use secure video- or web conferencing (Zoom; Zoom Video Communications) for the usability testing sessions due to COVID-19 pandemic restrictions, which prohibited in-person participant contact. Informed consent and a demographic and clinical information form (Multimedia Appendix 1) were obtained from participants before each iterative cycle. Participants were identified using a study ID, and data were stored on a secure OneDrive folder (Microsoft Corp) accessible only to the principal investigator and research officer. All participants were compensated with a user-identified CAD $25 (US $17.52) gift card.

### Study Design and Procedures

**Figure 2:**
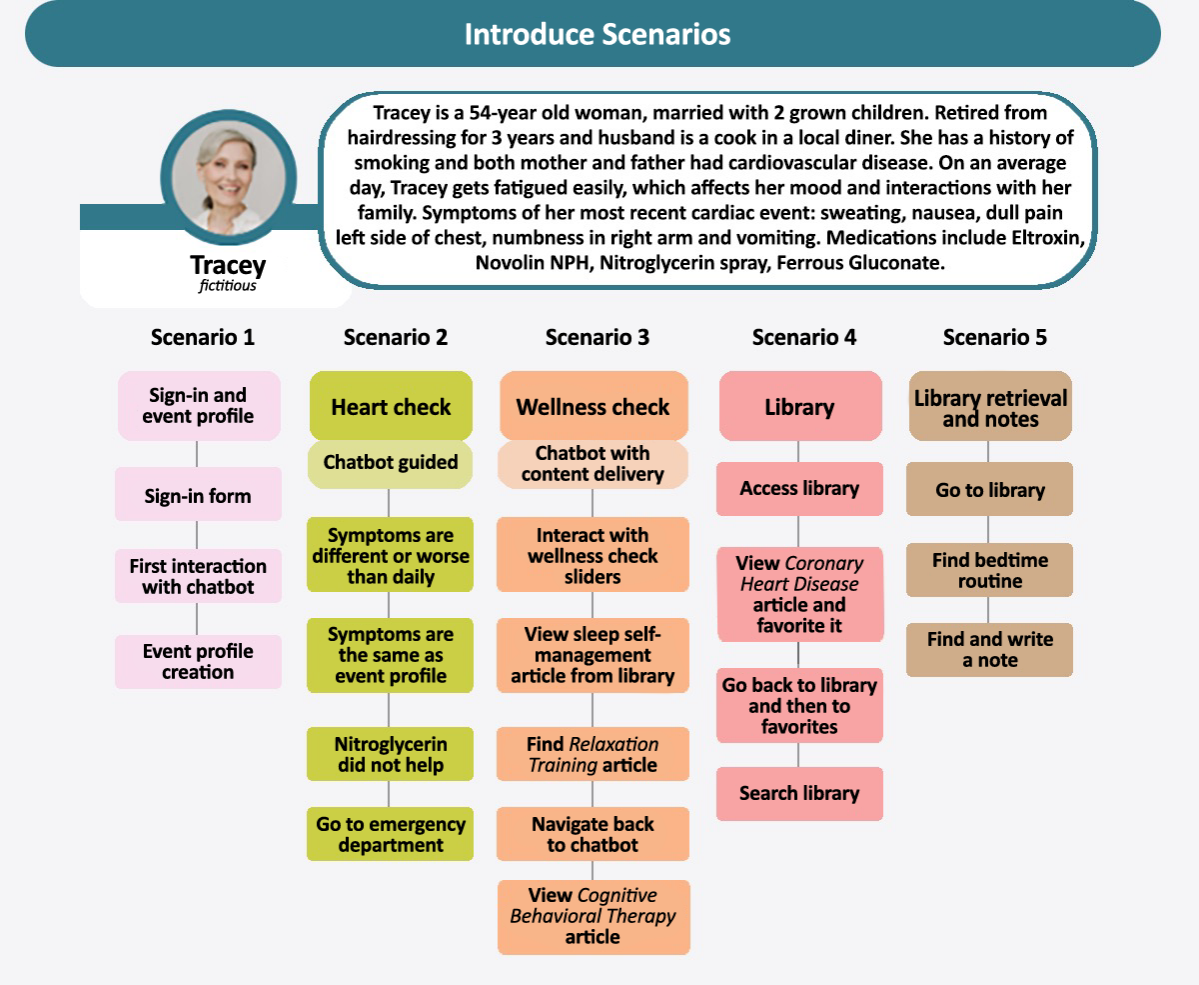
Usability testing workflow.

The usability of the *at heart* progressive web application focused on a think-aloud scenario-based approach to assess user performance (ie, ease of use, efficiency, and errors) and user satisfaction with *at heart's* content and functionality assessed qualitatively through the short scenario debrief questions and the interviews and quantitatively using the SUS [40]. This multi-method approach to usability testing is supported by a recent scoping review [41]. A sample of 10 eligible women was recruited to participate in the iterative usability testing cycles (n=5, 50% of the women participated in each of 2 testing cycles). This sample size was based on the experience of others [42-44], as well as recommendations that usability testing by 3 to 5 users can find approximately 85% of interface usability problems [45,46]. The first cycle tested user performance and satisfaction on a desktop computer, and the second cycle assessed user performance and satisfaction across various Android and iOS devices (ie, smartphones and tablets). Participants were provided with a brief explanation of the study and the *at heart* progressive web application before undergoing a 60- to 90-minute one-on-one observation period conducted and recorded through the Zoom videoconferencing platform. Participants were introduced to a case of a woman aged 54 years with symptoms that were “possibly cardiac” [47] and asked to sign in, complete an event profile (scenario 1), and then progress through a set of standardized scenarios that incorporated each core feature of *at heart*: Heart Check, Wellness Check, and the library (scenarios 2-5; Figure 2).

The “think-aloud” approach was used to capture users’ thought process and problem-solving as they progressed through *at heart's* core features in a systematic manner. After each scenario, the participants answered two short debrief questions: (1) “What did you like about the content and functionality of this specific scenario?” (2) “What did you dislike about the content and functionality of this specific scenario?”

In total, 2 investigators also recorded user comments and documented any problems encountered during each scenario using the usability testing error and efficiency documentation form (Multimedia Appendix 2). Three error types were captured on this form: (1) navigation errors (ie, failure to locate a function or follow the recommended screen flow), (2) presentation errors (ie, selection errors due to labeling ambiguities), and (3) control use errors (ie, improper entry field errors). Four semistructured interview questions were posed following the think-aloud scenarios to assess the overall satisfaction with *at heart's* content and functionality: (1) “What was your overall impression of the *at heart* progressive web application?” (2) “What did you like or dislike about the *at heart* progressive web application?” 3) “Is there anything that could be improved or changed?” (4) “Is there anything missing from the *at heart* progressive web application?” Participants then completed the SUS on the web using REDCap (Research Electronic Data Capture; Vanderbilt University), a secure web application. The SUS is a widely used scale that quantifies the usability of digital health applications. It consists of 10 questions with Likert scales that range from *strongly agree* to *strongly disagree*, and it has been validated across a range of interfaces, including web pages and web applications [40,48,49]. Scores range from 0 to 100, with an accepted benchmark mean SUS score of 68 (SD 12.5) [40]. One 7-point adjective-anchored Likert scale was added to the bottom of the SUS so that participants could rate the overall user-friendliness of *at heart* as *worst imaginable*, *awful*, *poor*, *ok*, *good*, *excellent*, or *best imaginable*. This adjective rating scale helps inform the absolute usability of a product [50-52], such as the *at heart* progressive web application. Numerical equivalents of 1 (*worst imaginable*) to 7 (*best imaginable*) were assigned to the adjectives for scoring. After the first cycle, revisions were made to the prototype, and the revised prototype was tested in a second usability testing cycle.

### At Heart Progressive Web Application

**Figure 3:**
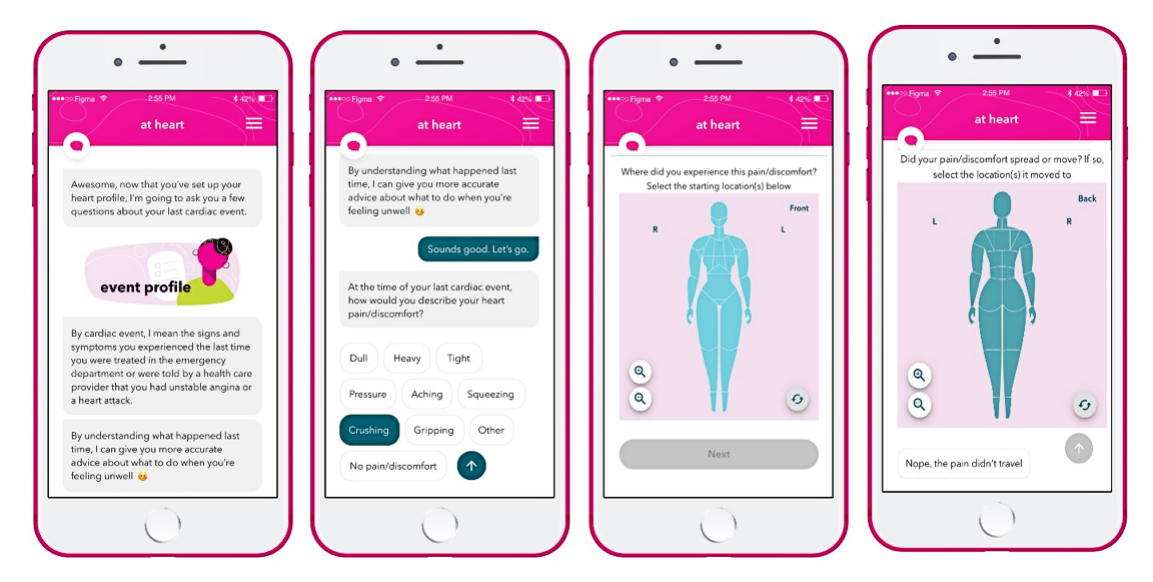
Screenshots of the *at heart* feature Event Profile prototype.

*At heart* is a novel progressive web application that consists of 3 core features: Heart Check, Wellness Check, and a library. Users are guided through the core feature set by a rule-based chatbot that manages content and conversations. An Event Profile is created on the initial log-in to the progressive web application (Figure 3). The Event Profile contains individualized or personalized data that include the quality and location of cardiac pain or associated symptoms experienced at the time of the participant’s last heart event (ie, treated in the emergency department). Female front and back full-body maps were specifically developed for *at heart* using the chest pain or associated symptom locations most commonly described in the literature [6,8,13,47,53].

Chatbot Holly asks women the same series of cardiac pain and symptom assessment questions in the Heart Check, which is programmed into the *at heart* progressive web application to occur every 3 days (Figure 2). The Heart Check can occur more frequently simply by asking chatbot Holly for a Heart Check. Rule-based symptom triage algorithms compare each Heart Check to the individually stored Event Profile to make recommendations on an appropriate level of care. Level of care recommendations are based on the similarity and timing of current cardiac pain or discomfort to that stored Event Profile, noting similarities between the current and previous descriptions of the quality (ie, heavy, tight, or pressure), location, radiation, and associated symptoms. Recommendations are assigned to one of three categories: (1) *red* or high risk (ie, similar or high-risk symptoms that have occurred within 24 hours), (2) *yellow* or moderate risk (ie, similar or high-risk symptoms that have occurred beyond 24 hours but within 7 days), and (3) *green* or low risk (ie, no similar or high-risk symptoms). Women who are categorized as *red* are encouraged to notify a family member of their current symptoms and call 911 (ie, to seek appropriate and urgent assessment of their symptoms). Women who are categorized as *yellow* are encouraged to see their primary care provider within 48 hours, and women who are categorized as *green* are permitted access to the *at heart* progressive web application. High-risk symptoms are defined as ≥3 new typical symptom features (ie, dull, heavy, or tight chest pain) [53] or any new associated symptoms, including shortness of breath; palpitations; a racing heart rate; or feeling lightheaded, faint, or dizzy [47].

**Figure 4:**
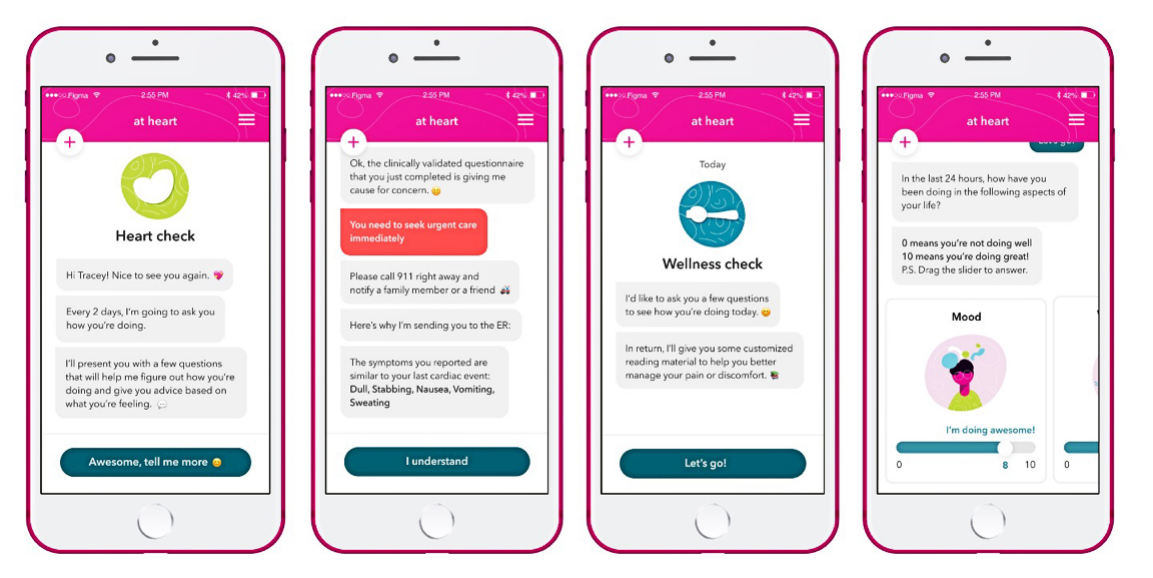
Screenshots of the Heart Check and Wellness Check features of the *at heart* prototype.

Women complete the Wellness Check, informed using features of the Brief Pain Inventory–Interference Subscale [54], to indicate the degree to which cardiac pain or associated symptoms interfere with 7 domains of life, which include general activities, paid and unpaid work, walking, mood, relations with others, sleep, and overall enjoyment of life (Figure 4). Wellness Checks are programmed to occur every 7 days at a minimum, and if the score in any domain is below a threshold, the chatbot uses rule-based algorithms to deliver educational content from the library.

The library contains scientific papers, each with a lay summary, covering educational content about CAD in women, with self-management advice to promote good health and well-being [29]. The library also contains videos and podcasts from women with lived experiences of CAD. *At heart* was evaluated by Health Canada and deemed not to be a medical device as defined in the Guidance Document: Software as a Medical Device (SaMD): Classification Examples [55]. *At heart* provides self-management support to women who have CAD and, through a chatbot, guides women to the most appropriate form of assessment (ie, primary care and emergent care) based on their medical symptoms.

### Data Analysis

Quantitative descriptive data from the demographic and clinical information form and the SUS were analyzed using SPSS Statistics (version 29; IBM Corp). Debrief feedback from each of the 5 scenarios with data from the usability testing error and efficiency documentation form was reviewed after each of the 2 iterative usability testing cycles. Conventional content analysis as outlined by Sandelowski [56] was used to analyze qualitative data obtained from the interview questions. In total, 2 researchers independently assigned codes to label key actions, thoughts, and ideas. Related codes were grouped into categories, and any disagreements were discussed between the 2 researchers. A list of prototype issues was generated and prioritized based on risk to patients and ease of improvement [57]. Modifications were communicated to the website design and development team. Prototype changes made after the first cycle were evaluated in the second cycle, and prototype changes following the second cycle were completed before the phase-3 RCT.

## Results

### Participant Characteristics

A total of 10 women meeting the eligibility criteria were invited to participate in the usability testing from March 31, 2020, to April 17, 2020 (cycle 1), and from November 17, 2020, to November 30, 2020 (cycle 2). Women across the usability testing cycles had a mean age of 55.6 (SD 7.3) years, and most had a diploma (5/10, 50%), an undergraduate degree (2/10, 20%), or a graduate degree (2/10, 20%). Most of the women were employed full (3/10, 30%) or part time (2/10, 20%) and earned >CAD $70,000 (US $48,881.80) annually (6/10, 60%); 50% (5/10) lived with a disability, 50% (5/10) were the primary household earners, and 80% (8/10) were the primary persons responsible for housework in the home. The women reported cardiac pain or associated symptoms for 1 to 2 years (3/10, 30%), 2 to 5 years (3/10, 30%), or >5 years (3/10, 30%). The most common comorbid conditions included depression (4/10, 40%) and anxiety (4/10, 40%), and 40% (4/10) of the women had had a previous myocardial infarction. Participant demographics by usability testing cycle are reported in Table 1.

All women (10/10, 100%) had a computer at home and were comfortable (3/10, 30%) or very comfortable (7/10, 70%) using a computer, comfortable (1/10, 10%) or very comfortable (9/10, 90%) using the internet, and comfortable (3/10, 30%) or very comfortable (7/10, 70%) using smartphone or tablet apps. Participants’ comfort levels and use of the computer, the internet, and smartphone or tablet apps are reported in Table 2. All women selected their own devices for usability testing. Women in the first cycle used only the Google Chrome browser (5/5, 100%), and women in the second cycle used an iPad mini version 9.3.5 (1/5, 20%), Samsung Galaxy S10e (1/5, 20%), iPhone 8 (2/5, 40%), and iPhone 11 (1/5, 20%).

**Table 1:** Participant characteristics.

Characteristic			Cycle 1 (n=5)		Cycle 2 (n=5)
Age (y), mean (SD)			58.4 (4.827)		52.8 (8.872)
Gender identity (woman), n (%)			5 (100)		5 (100)
Indigenous, n (%)			0 (0)		0 (0)
Identifying as a visible minority, n (%)			1 (20)		1 (20)
Living with a disability, n (%)			2 (40)		3 (60)
Gendered roles (housework hours), mean (SD)			6.6 (5.1)		10.2 (8.0)
Primary person responsible for housework, n (%)			4 (80)		4 (80)
**Educational level, n (%)**					
	High school	0 (0)		1 (20)	
	Diploma	2 (40)		3 (60)	
	Bachelor’s degree	2 (40)		0 (0)	
	Master’s degree	1 (20)		1 (20)	
**Employment status, n (%)**					
	Full time	1 (20)		2 (40)	
	Part time	1 (20)		1 (20)	
	Unemployed	3 (60)		2 (40)	
**Income, n (%)**					
	<CAD $15,000 (US $10,474.70)	0 (0)		1 (20)	
	CAD $15,000-$29,900 (US $10,474.70-$20,879.50)	0 (0)		0 (0)	
	CAD $30,000-$49,900 (US $20,949.40-$34,845.80)	0 (0)		1 (20)	
	CAD $50,000-$69,900 (US $34,915.60-$48,812)	1 (20)		0 (0)	
	CAD $70,000-$99,900 (US $48,881.80-$69,761.40)	3 (60)		1 (20)	
	>CAD $100,000 (US $69,831.20)	1 (20)		1 (20)	
	Not reported	0 (0)		1 (20)	
Primary earner, n (%)			1 (20)		4 (80)

**Table 2:** Use of the computer, the internet, and smartphone or tablet apps.

Characteristic		Cycle 1 (n=5)	Cycle 2 (n=5)
Computer use—home, n (%)		5 (100)	5 (100)
Computer use—work, n (%)		2 (40)	3 (60)
Computer hours per week, mean (SD)		6.4 (2.302)	8 (0.000)
**Comfortable using computers, n (%)**			
	Comfortable	1 (20)	2 (40)
	Very comfortable	4 (80)	3 (60)
Internet use, n (%)		5 (100)	5 (100)
Internet hours per week, mean (SD)		6.8 (2.168)	8 (0.000)
**Comfortable using the internet, n (%)**			
	Comfortable	1 (20)	0 (0)
	Very comfortable	4 (80)	5 (100)
Smartphone or tablet app use, n (%)		5 (100)	5 (100)
Smartphone or tablet app hours per week, mean (SD)		6.4 (2.302)	7 (1.414)
**Comfortable using smartphone or tablet apps, n (%)**			
	Comfortable	1 (20)	2 (40)
	Very comfortable	4 (80)	3 (60)

### User Performance

User performance was assessed through ease of use, efficiency, and observation of testing errors (ie, navigation, presentation, and control use errors) through each scenario and cycle of usability testing. Testing errors included those related to navigation (difficulties moving through or locating content), presentation (selection errors due to labeling), or control use (improper entry field errors) across the 5 scenarios in 2 usability testing cycles (Figure 2). In scenario 1 of cycle 1 (sign-in, chatbot, and Event Profile), participants reported a low contrast between the text and the background and small font at sign-in. Participants also wanted clarification that the chatbot was not a real person. These were flagged as high priority, and subsequent revisions included larger font and chatbot and user text boxes identified using different colors, and a page was added to the *at heart* progressive web application to describe the chatbot, including its development and function. Specific text included the following: “let me tell you a little more about myself. I’m an educated chatbot, thoughtfully designed by a group of women’s heart health experts. I’ll be here to help you understand more about your heart pain/discomfort through regular check-ins, and I’ll send you tips and tricks for managing that pain. If you want to learn more about my makers, the same ones who named me Holly, go to the menu above...” A summary of suggestions and subsequent changes made to the progressive web application during usability testing cycle 1 is shown in Table 3.

The scenarios were repeated in the second cycle to assess user performance and user satisfaction across various Android and iOS devices (ie, smartphones and tablets). In scenario 1 of cycle 2 (sign-in, chatbot, and Event Profile), participants requested clarification on the terminology used in some of the questions at sign-in, so descriptors and more answers were added to these questions. Participants also had difficulty choosing specific areas on the body map, so the body maps were subsequently amended. In scenario 2 of cycle 2 (chatbot-guided workflow for the Heart Check), participants appeared confused with the order and speed of the questions delivered by the chatbot. The chat time stamp was subsequently increased for desktop, Android, and iOS devices. This allowed women ample time to read and respond appropriately to the Heart Check questions, especially those with longer messages and prompts. Finally, the Heart Check button was reported to be too small and gray, so the size and contrast were adjusted. The chatbot-guided workflow for the Wellness Check (scenario 3, cycle 2) required minor adjustments to ensure that the chatbot selected appropriate resources from the Wellness Library based on wellness scores of ≤4. One participant suggested that “anaphylaxis” be added to the Share My Data section and related articles be added to the library. As a result, lay summaries were generated, and scientific papers were added to the library for Kounis syndrome (n=3) and mast cell activation syndrome (n=2). Kounis syndrome is a complex multisystem arterial condition caused by mast cell activation and T-lymphocyte and macrophage interactions [58]. The resulting allergic, hypersensitivity, and anaphylactic reactions cause coronary symptoms (ie, cardiac pain), and this is referred to as Kounis syndrome [59]. A summary of suggestions and subsequent changes made to the progressive web application during usability testing cycle 2 is also shown in Table 3.

**Table 3:** Summary of suggestions and subsequent changes during usability testing.

Suggestions				Changes
**Cycle 1 (n=5)**				
	**Scenario 1—sign-in, chatbot, and Event Profile**			
		There was a low contrast between the text and the background and small font at sign-in.	Larger font and chatbot and user text boxes identified using different colors were implemented.	
		Clarification that the chatbot is not a real person was requested.	A page was added to the *at heart* progressive web application to describe the chatbot, including its development and function.	
		Participants selected only 1 descriptor on the body map and reported lack of clarity that more than one descriptor could be selected.	Directions for completing the body map were enhanced.	
	**Scenario 2—Heart Check**			
		None	None	
	**Scenario 3—Wellness Check**			
		None	None	
	**Scenario 4—library**			
		Participants had difficulty locating the Favorites section of the library.	The Favorites section was moved to the top of the screen and identified with a larger icon that represented a heart.	
	**Scenario 5—library retrieval and notes**			
		None	None	
	**Other**			
		None	None	
**Cycle 2 (n=5)**				
	**Scenario 1—sign-in, chatbot, and Event Profile**			
		Participants requested clarification on the terminology used in some of the questions at sign-in.	Descriptors and more answers were added to the questions for clarity.	
		Participants had difficulty choosing specific areas on the body map to describe their symptoms.	The body maps were subsequently amended to include axillae and body map descriptors.	
	**Scenario 2—Heart Check**			
		Participants appeared confused with the order and speed of the questions delivered by the chatbot.	The chat speed was reduced for desktop, Android, and iOS devices.	
		The Heart Check button was reported to be too small and gray.	The size and contrast of the “Heart Check” button were adjusted.	
	**Scenario 3—Wellness Check**			
		The chatbot says the following: “based on what you’ve told me, here are some topics...” However, the chatbot presented all topics even for wellness scores that were excellent (ie, the readings from the library did not appear tailored to the needs of each participant).	Minor adjustments were made to the rules to ensure that the chatbot selected appropriate resources from the wellness library based on wellness scores of ≤4.	
	**Scenario 4—library**			
		None	None	
	**Scenario 5—library retrieval and notes**			
		None	None	
	**Other**			
		Add anaphylaxis to the Share My Data section.	This was added.	
		Add anaphylaxis articles to the library as they related to women and heart disease.	Lay summaries were generated and scientific papers were added to the library for Kounis syndrome (n=3) and mast cell activation syndrome (n=2).	
		Participants requested to have a more robust Share My Data section, with opportunity to record medications and other comorbid conditions.	The introduction and the content of the Share My Data section were enhanced.	

### User Satisfaction

#### Overview

User satisfaction was assessed using the SUS and the 4 semistructured interview questions. Overall, 10 participants across 2 cycles of testing reported the overall usability of the *at heart* progressive web application as highly acceptable (mean SUS score 81.75, SD 10.41). There was no difference in SUS scores between cycle 1 (mean SUS score 81.5, SD 8.59) and cycle 2 (mean SUS score 82.0, SD 13.04; *t*_8_=−0.72, 95% CI −16.6 to 15.6). A total of 90% (9/10) of the participants rated the user-friendliness of *at heart* as good or excellent. All participants (10/10, 100%) thought that *at heart* was easy to use (SUS question 3; ie, most women could learn to use *at heart* very quickly):

I think everything was pretty straightforward. It was overall really, really good.

Cycle 2; participant 3

None of the participants indicated that they would need the support of a technical person to be able to use *at heart* (SUS question 4), and 80% (8/10) of the participants found the various functions in *at heart* well integrated (SUS question 5) with minimal inconsistencies (SUS question 6):

Well...I was a bit worried it might be confusing, but it’s not confusing at all. It’s very intuitive, it, you’ve built it so it’s similar to a lot of other apps out there, right? So, clicking here, you know, submitting the button. So, it seems to be pretty normal as far, it looks, the same look and feel as other apps that are out there which are good. It seems to be easy. I’m not a tech person, so I wanted to make sure that—I was nervous that I wasn’t going to be able to manage it because I, I’m not great with technology, but it was easy. So, yeah. I mean, I think it’s a great tool.

Cycle 2; participant 5

A total of 8 themes emerged from the interview data (ie, engaging, comprehensive, understandable, credible, relevant, affirming, personalized, and innovative) and can be found in Multimedia Appendix 3.

#### Engaging

Participants commented on the layout, visual appeal, language, and name and logo of the progressive web application. They found the content easy to read and maneuver. Important content was highlighted, and participants reported this to be helpful. Article summaries were presented in larger font and used lay language. At the outset of this study, the progressive web application was named HEARTPA♀N [29]. Participants recommended a name change to one that was less focused on pain as many women describe their cardiac pain as discomfort and have other associated symptoms (eg, dyspnea and fatigue). They felt that the name *at heart* was more personally welcoming and less focused on pain. The heart logo was simple yet distinctive and recognizable.

#### Comprehensive

Participants indicated that the progressive web application provided necessary information for helping them make decisions about their cardiac symptoms. They liked that it was specific to women and that they could search through the library to improve their knowledge about wellness and women’s heart health. Participants also commented that the chatbot assisted them in making decisions:

...the bot kind of identifies for me what the big blind spots are that I am perhaps not noticing.

#### Understandable

Participants indicated that *at heart* was intuitive, the content made sense, and it flowed well. The tone and the language were suitable and clear. Participants commented on the appropriateness of the associated symptom descriptors included in the Event Profile and Heart Check.

#### Credible

Participants liked that *at heart* was developed as a living progressive web application. They particularly liked the breadth and depth of scientific articles and lay summaries included in the *at heart* library. Participants valued having an evidence-informed digital health self-management program specifically designed for women with CAD living with cardiac pain and associated symptoms.

#### Relevant

Participants commented on the relevance and accessibility of content in the *at heart* library. The chatbot asked relevant questions and was able to use rule-based algorithms to provide advice to women about timely assessment in the emergency department. They particularly noted the relevance of having their Event Profile and Heart Check information stored so that they could refer to it when visiting their health care providers. The content was applicable to their everyday lives.

#### Affirming

Participants looked for affirmation from *at heart's* chatbot when they were having cardiac symptoms. They indicated that they would have otherwise consulted with family members or Facebook group members for guidance. Affirmation of symptoms was important to women, and they appreciated the high-alert messages to seek urgent care immediately.

#### Personalized

Participants liked the chatbot. They found it to be personal, immediately interactive, accessible, calming, and friendly. They commented that the chatbot may help make them feel less alone if they went to the emergency room because it checked in with them to see how they were doing. The chatbot provided comfort at times when participants felt scared. Participants also valued the videos and podcasts as they also helped them feel that they were not alone in their experience with heart disease.

#### Innovative

Participants felt that *at heart* provided an opportunity for further research focused on women’s heart health. They were willing to share anonymized data on socioeconomics, risk factors, gynecological or obstetrics history, lifestyle, medications, and other conditions (eg, arthritis or depression) to improve outcomes for women at risk of or with heart disease. Participants also valued the ability to journal and take notes in the progressive web application. They valued the opportunities that *at heart* could provide to women who live in more isolated areas or those who live alone. The *at heart* progressive web application was described as all-encompassing, covering necessary content to improve knowledge and decision-making, with a chatbot to ask questions and deliver information in a comforting manner.

## Discussion

### Principal Findings

There is growing support for the importance of usability testing for mobile health and eHealth innovations [60-62]. Usability testing incorporates an iterative process of testing and refining to meet end-user needs [63]. The objectives of this study were to assess the user performance (ie, ease of use, efficiency, and errors) and user satisfaction (SUS) of a progressive web application for women with cardiac pain. User performance was assessed through ease of use, efficiency, and observation of testing errors (ie, navigation, presentation, and control use errors) through 5 brief scenarios and 2 cycles of usability testing.

The overall usability of the *at heart* progressive web application was rated highly by 10 participants who completed the usability testing cycles; the progressive web application was easy to use and efficient. However, 2 high-priority testing errors were identified during the usability testing cycles. The first involved small font at sign-in and low contrast between the text and background, and these were corrected by enlarging the font and making alterations to the text and background colors. The second was to clarify that the chatbot was not a real person. A significant amount of time was committed to addressing potential language challenges during the development of the chatbot, and there were no technical, design, or language challenges identified during the usability testing cycles. In fact, the design and language or conversation of the chatbot appeared to mimic human-to-human interaction so closely that participants requested confirmation that the chatbot was actually nonhuman.

The *at heart* chatbot is a simple rule-based conversational agent designed to mimic human-to-human interaction at sign-in and creation of an Event Profile and during completion of the Heart Check and Wellness Check. Our previous systematic review and meta-analysis indicated that self-management programs were more effective in reducing cardiac pain and associated symptoms (eg, dyspnea and fatigue) in women if they included collaboration and support from health care providers [33]. Chatbots are conversational agents able to promote health and provide education and support [64]. Their use in health care is still in a developmental stage; they could improve access to care and health care provider–patient communication, but more evidence is needed. Technical, design (ie, lack of empathy), and language challenges [65] can impede the integration of chatbot technologies into health care [64]. Adopting user-centered and theory-based designs, optimizing user experiences, and addressing patient concerns can improve chatbot uptake and use [64]. Our chatbot relies on scripted computational algorithms, with specific rules for its text-based conversations [66]. More advanced human-machine conversation is now based on natural language processing and large language models that use artificial intelligence methods to learn, understand, and produce structured language content [66,67]. Health care delivery is rapidly changing and is driven by social, scientific, and technological change; our future chatbot may need to emulate person-to-person conversation through dialogue and body movements, with appropriate expressions of empathy and compassion [68]. We are really at a cusp in health care; growth in chatbot use will be driven by a desire for health and wellness and 24-hour access to care, with a growing number of platforms from which to build an intelligent and emotive chatbot in the future [69].

*At heart* is the first of its kind; no smartphone- or web-based self-management program has been co-designed and systematically developed with women who have lived experience (phase 2A) and then tested with women who have cardiac pain (phase 2B). We used the individual and family self-management theory [70,71], mobile device functionality, and the pervasive information architecture of mobile health interventions [72] and followed the MRC’s guidance for developing complex interventions [30-32]. *At heart* was designed as a progressive web application aimed at delivering nativelike user experiences regardless of the browser or the mobile device [73]. This study identified that the comprehensiveness and credibility of the information was important to women. The information helped them understand more about CAD and wellness and provided them with guidance in decision-making. At inception, women clearly articulated the need for a web application that was accessible across Android and iOS operating systems, including computers, smartphones, and tablets. In phase 1, women identified the need to access the library anytime and anywhere (eg, while waiting for dental appointments). As women were involved at the outset in co-design, no other significant user performance or satisfaction–related issues were identified. Female front and back full-body maps were specifically developed for *at heart* using the chest pain or associated symptom locations most commonly described in the literature [6,8,13,47,53]. These required only minor refinements during usability testing (ie, more precise identification of the axillae). Importantly, participants also viewed *at heart* as a way to contribute to the future of women’s heart health. Women feel “stopped at the gate,” and they want to take charge and advocate for better awareness, education, diagnosis, and management [74].

### Limitations and Strengths

First, usability testing was conducted on a predominantly affluent sample of participants, which limits the generalizability of our results. A total of 60% (6/10) of the participants who took part in usability testing earned >CAD $70,000 (US $48,881.80) annually. They were also well educated, and most (8/10, 80%) were White. Canada is known for its diversity in race and ethnicities— over 450 ethnic or cultural origins were reported in the 2021 census [75]. A total of 1 out of 5 people in Canada is born elsewhere; the 3 largest visible ethnic groups are South Asian, Chinese, and Black individuals, representing 60% of the Canadian racial and ethnic populations [76]. Although this was a limitation in the sample for the usability testing, this is not a limitation specific to the *at heart* progressive web application. In fact, the *at heart* library contains heart disease and heart wellness scientific papers and lay summaries related to South Asian and Indigenous people. These include risk and traditional practices such as drumming and healing and talking circles. However, it will be necessary to include a more diverse sample of women that encompasses those in less populous areas where age-standardized cardiovascular-related deaths in women are highest [77]. It will also be important to target recruitment of women from South Asian and Afro-Caribbean races and ethnicities, those with disabilities, those of a lower socioeconomic status, and those across age and gender identities who also experience greater risk of cardiovascular disease [77-79].

In addition, heterogeneous use of platforms and devices may also be a limitation to the usability testing. Progressive web applications are designed to work across platforms, eliminating the cost, fragmentation, and need to develop the same application several times for multiple platforms. They are designed to behave like mobile apps, working seamlessly across Android and iOS devices. However, past research indicates that progressive web applications may not be as smooth as mobile apps on Android devices [80]. Our sample size was limited, and only 10% (1/10) of the participants used an Android device for testing. Further research is needed to evaluate the usability of the *at heart* progressive web application across various platforms and devices. The phase-3 pilot RCT will determine the feasibility of implementing the *at heart* intervention, including the *feasibility* of randomization, recruitment, and retention; *acceptability* and barriers to implementing the intervention; and the extent of engagement with the intervention. The phase-4 effectiveness-implementation hybrid trial (type I) will investigate the effectiveness and implementation of *at heart* among women living in Canada using the practical, robust implementation and sustainability model [81]. The MRC [30-32] discusses 4 phases of complex intervention research (eg, development, feasibility, evaluation, and implementation) and the importance of refinements with each phase transition depending on the context [70,71] in which the intervention is evaluated and implemented. The practical, robust implementation and sustainability model [81] aligns contextual needs with intervention design using an inequity, systems thinking, and cocreation engagement approach. Context will be addressed by considering the individual perspectives on the intervention, external environment, characteristics of those receiving the intervention, and supports and resources needed to deliver the intervention [81]. Recruiting representative populations can be improved through strengthened community partnerships in governance and decision-making [82-84], and this will be a focus in subsequent phases of this research.

Second, remote usability testing methods were used during the COVID-19 pandemic. Testing a technology’s usability remotely has inherent advantages as well as limitations. While participants can prefer remote testing to in-person testing, it can take longer for participants to complete tasks during remote testing, more errors can be made compared to in-person testing sessions, and context and indirect cues can be missed during remote testing sessions [85]. However, the approach taken to usability testing in this study was iterative, comprehensive, and aligned with methods used by others recently reported in a scoping review [41]. Of 133 articles included in this scoping review, 105 used questionnaires, 45 used a think-aloud approach, and 37 used interviews in their usability testing methods [41]. Moreover, many studies used a combination of 2 (n=46) or 3 (n=30) methods of testing, and the SUS was the most reported and validated questionnaire used to assess satisfaction and usability [41]. Finally, the *at heart* chatbot is rule based and designed for specific functions within the progressive web application. It is not led by artificial intelligence technologies (ie, natural language processing or large language models) and, therefore, may have inherent limitations that were not identified during the 2 cycles of usability testing (ie, personality, flexibility, dialogue structure, and conversation complexity or flow) [65].

### Conclusions

This study does provide initial support for the *at heart* progressive web application for women with cardiac pain. Participants rated the performance of and satisfaction with this progressive web application as high. Ongoing evaluations in phases 3 and 4 should aim to examine the feasibility and acceptability and the extent of engagement with the *at heart* core feature set: Heart Check, Wellness Check, and the library. In addition to assessing effectiveness in an effectiveness-implementation hybrid trial (type I), describing and better understanding the context for implementation (eg, race and ethnicity and geography) will be necessary.

## Appendix

Multimedia Appendix 1 Participant demographic and clinical information form.

Multimedia Appendix 2 Usability testing error and efficiency documentation form.

Multimedia Appendix 3 Summary of participant comments by theme.

## Abbreviations

CAD: coronary artery disease
HRQoL: health-related quality of life
MRC: Medical Research Council
RCT: randomized controlled trial
REB: Research Ethics Board
REDCap: Research Electronic Data Capture
SMD: standardized mean difference
SUS: System Usability Scale
