# Supplementary material for: Development and Systematic Evaluation of a Progressive Web Application for Women With Cardiac Pain: Usability Study
Source: JMIR Hum Factors. 2025 Apr 17;12:e57583. doi: 10.2196/57583 (PMC12046265; doi:10.2196/57583)
Supplement: Multimedia Appendix 1 [file humanfactors_v12i1e57583_app1.pdf]

Multimedia Appendix 1

**Participant Demographic and Clinical Information Form**

*at heart* ID:

Date (*year-month-day*):

This information will be summarised in order to describe the sample. **No individual information will be shared. Your name is not on this questionnaire and will not be linked with your answers.**

A1. What is your Date of Birth?

_______________________________________________

Month Day Year

A2. Please select your preferred gender identity. You may select more than one:
___ Woman ___ Gender-fluid ___ Non-binary ___ Two-spirit
___ Prefer not to answer

A3. Do you identify as Indigenous, that is First Nations (North American Indian), Metis, or Inuit?
___ Yes ___ No
___ Prefer not to answer

A4. Do you identify as a member of a visible minority in Canada?
___ Yes ___ No
___ Prefer not to answer

A5. Are you a person with a disability?
___ Yes ___ No
___ Prefer not to answer

A6. Please mark your highest educational level:
___ $_1$Elementary school ___$_2$ High school ___$_3$ Diploma/Certificate
___ $_4$Bachelors Degree ___$_5$ Master's Degree ___$_6$ PhD Degree

A7. What is your current employment status?
___ $_1$ Full-time ___ $_2$ Part-time ___ $_3$ Unemployed
___ $_4$ Retired

A8. How many years have you had cardiac pain?
___ $_1$ 3 to 6 months ___$_2$ 6 months to 1-year ___$_3$ 1 to 2 years
___ $_4$ 2 to 5 years ___$_5$ More than 5 years

A9. Have you ever had a coronary angiogram?
___ $_1$ Yes ___ $_2$ No

A10. Have you ever had a percutaneous coronary intervention (PCI)/Stent?

___ 1 Yes ___ 2 No

A11. Have you ever had coronary artery bypass graft surgery?
___ 1 Yes ___ 2 No

A12. What other conditions have you had/do you have?

| | | |
|---|---|---|
| ___ 1 Stroke | ___ 2 Osteoporosis | ___ 3 Asthma |
| ___ 4 Depression | ___ 5 Arthritis | ___ 6 COPD |
| ___ 7 Neurological Disease | ___ 8 Upper GI Disease | ___ 9 Anxiety |
| ___10 Hearing Loss | ___11 Visual Impairment | ___12 Diabetes |
| ___13 Heart Attack | ___14 Heart Failure | ___15 PAD |

A13. Do you use a computer at home?
___ 1 Yes ___ 2 No

A14. If you have outside paid employment, do you use a computer at work?
___ 1 Yes ___ 2 No

A15. What is your comfort level on the computer?
___ 1 Not at all comfortable
___ 2 A little comfortable
___ 3 Comfortable
___ 4 Very comfortable

A16. Approximately how many hours do you spend on a computer per week?
___ 1 0 ___ 2 1-2 ___ 3 2-3 ___ 4 3-4 ___ 5 4-5 ___ 6 5-6 ___ 7 6-7
___ 8 More than 7

A17. Do you use the Internet at home?
___ 1 Yes ___ 2 No

A18. What is your comfort level on the Internet?
___ 1 Not at all comfortable
___ 2 A little comfortable
___ 3 Comfortable
___ 4 Very comfortable

A19. How many hours do you spend on the Internet per week?
___ 1 0 ___ 2 1-2 ___ 3 2-3 ___ 4 3-4 ___ 5 4-5 ___ 6 5-6 ___ 7 6-7
___ 8 More than 7

A20. Do you use Smartphone apps?
___ 1 Yes ___ 2 No

A21. What is your comfort level using Smartphone apps?

___ 1 Not at all comfortable
___ 2 A little comfortable
___ 3 Comfortable
___ 4 Very comfortable

A22. How many hours do you spend on a Smartphone app per week?
___ 1 0 ___ 2 1-2 ___ 3 2-3 ___ 4 3-4 ___ 5 4-5 ___ 6 5-6 ___ 7 6-7
___ 8 More than 7

A20. Please indicate if you are interested in participating in future *at heart* research?
___ 1 Yes ___ 2 No
