# Supplementary material for: Development and Systematic Evaluation of a Progressive Web Application for Women With Cardiac Pain: Usability Study
Source: JMIR Hum Factors. 2025 Apr 17;12:e57583. doi: 10.2196/57583 (PMC12046265; doi:10.2196/57583)
Supplement: Multimedia Appendix 2 [file humanfactors_v12i1e57583_app2.pdf]

Multimedia Appendix 2

Usability Testing Error and Efficiency Documentation Form

Cycle 1: *at heart* web application ____________________________
browser

1. Start time: _____________

2. End time: _____________

3. Total time to complete tasks: _____________

4. Did participant view all required **elements of SITE**? YES NO
   If NO, reason: ____
   ____
   ____

**Type of Error**	**Number of errors made**	**Length of time to recovery**	**Not recovered/ fatal error**
Navigation errors (difficulties moving through or locating content)			
Presentation errors (selection errors due to labelling)			
Control usage problems (improper entry field errors			

Multimedia Appendix 2

Cycle 2: Android/iOS: ___________________________
device

1. Start time: ______________

2. End time: ______________

3. Total time to complete tasks: ______________

4. Did participant view all required **elements of APP**? YES NO
If NO, reason:____
____
____

**Type of Error**	**Number of errors made**	**Length of time to recovery**	**Not recovered/ fatal error**
Navigation errors (difficulties moving through or locating content)			
Presentation errors (selection errors due to labelling)			
Control usage problems (improper entry field errors			
