# Supplementary material for: Development and Systematic Evaluation of a Progressive Web Application for Women With Cardiac Pain: Usability Study
Source: JMIR Hum Factors. 2025 Apr 17;12:e57583. doi: 10.2196/57583 (PMC12046265; doi:10.2196/57583)
Supplement: Multimedia Appendix 3 [file humanfactors_v12i1e57583_app3.pdf]

Table S1 *Summary of participant comments by theme.*

| Theme | Quotes |
|---|---|
| Engaging—layout, visual appeal, language, and name and logo | • "So good. And even just looking at the format...it makes it really easy to read—the way it's spaced out and with the bullets and stuff." [Cycle 2; participant 3]<br>• "I like the website. I find that the, just the layout of it is easy to maneuver...I can read it just the way it's laid out...A lot of people will walk away from an app...if they get frustrated because it's too hard to figure out. No, this this seems quite laid out very nicely." [Cycle 2; participant 3]<br>• "It's highlighted in red, so it's hard to miss." [Cycle 1; participant 1]<br>• "[Library] Well, this was helpful...I like how they're [lay summaries] nice and big and then they're bold..." [Cycle 2; participant 3]<br>• "[at heart]...I like the name...I like that it's not pointing at pain, but I like the simplicity of the name [at heart]. The other name that I really like that's in Canada is 'heart life.' There's just something about both of those phrases that just, I don't know, they just feel nice and they don't feel too sterile. I like the logo heart, too. It's a nice simple one, but it's very distinctive enough that you can recognize it. So, that was well done." [Cycle 2; participant 1]<br>• "[at heart] I like that it's a heart focused one...but this makes me feel like I'm personally welcome. Because it's specific to heart and it's getting at the heart of the matter, because I like that play on words. And that's my heart. How's my heart doing? And I like that you guys are moving away from the pain language." [Cycle 2; participant 1] |
| Comprehensive—contains necessary information and assists in decision-making | • "I mean, I like this in being able to choose what I read. This way I decide what my problem is. The other way, the bot kind of |

| | |
|---|---|
| | identifies for me what the big blind spots are that I am perhaps not noticing." [Cycle 1; participant 1]<br>• "I feel like, how this does talk about 'what are your symptoms' and then they'll give you information based on how I'm feeling. I like that because sometimes I don't know what this means when I'm feeling this. And I don't want to tie up the medical system and go to emerg every time...So, you do have to be self-independent and try to figure things out yourself sometimes. So, I do like how this website is, gives me information. And you know, it says, 'go 911.' Absolutely. That's good." [Cycle 2; participant 3]<br>• "...I like the explanation about, you know, heart pain. And it's different depending...maybe I feel my heart fluttering and that's more of just a discomfort...but it maybe, it's not painful. So that helps, that helps. You say OK, so include everything, like think about all the different things that might be happening in your body when you talk about your heart." [Cycle 2; participant 5]<br>• "I think people will be happy that there's something built for women. I think it actually calls out the items and the symptoms that are specific to women and I think it will help people and women understand that feeling heartburn might be an issue and it is a symptom...it might help you understand the symptoms for a heart attack are very different than they are for a man. And that you should think about all of those if you are experiencing some sort of a pain...I've seen people on...some of the Facebook groups say, '...You know, I'm having this issue, and I don't know what to do. Should I go to the emergency?' And, people are responding back to them, 'Go to emergency right away.' And to me that's more stressful...So, if we can get this in front of them, that's what they're looking for, right. 'Here's my symptoms. Here's what I'm feeling. Oh, OK. This is, this bot, this is now telling me...I should be |

| | |
|---|---|
| | concerned.' So, I'm going to go. I think I would, I would rely more on an app than I would of all these people...This is giving you some clear understanding of symptoms, what possibly you should do, and when it seems to be an issue that you should be looking into. So, I think it's, it's helpful." [Cycle 2; participant 5]<br>• "[Library] I am always curious, so I definitely would look through the library because I like to, I like to explore different options and do reading. I like to find—I definitely want to know more about my condition and stuff, right?" [Cycle 2; participant 3] |
| Understandable—readability and lay language | • "No, no I think that it is very intuitive. It makes sense to me and it reads well, it flows well, and I think anybody would understand it." [Cycle 1; participant 2]<br>• "[Chatbot] It [tone/language] sounds good." [Cycle 2; participant 4]<br>• "Yeah, I think that's clear. You explained the cardiac event, what it, when, you know, the signs, the symptoms the last time you were in the hospital or treated in the emergency department or told that you were having. Yeah, I think it's fair. I think it's a good question." [Cycle 2; participant 5]<br>• "I'm thinking about when I actually had my heart attack right, and I'm starting to think about what were my symptoms at the time. And I think you've got some good words here. I had no pain in my heart during my heart attack. So, you know you have that category there, which is good..." [Cycle 2; participant 5]<br>• "I think the question [about last cardiac event] makes sense. I think it's fair with the options that you've provided." [Cycle 2; participant 5] |
| Credible—accurate and trustworthy | • "I mean, I would probably feel a little bit more justified in going with having been encouraged by the app because, you know, we're not |

| | |
|---|---|
| | making stuff up here, right? We're doing something that's more clinically supported, you know? So, as a user, I would feel more inclined to go [seek medical care]." [Cycle 1; participant 4]<br>• "I feel comfortable that what it's saying to me is actually, reasonably well-researched and were going to be reliable information." [Cycle 1; participant 4]<br>• "And I like that this is one of those things where I can see...it will be immediately helpful, but at the same time, after you've gotten feedback for a year, there will be even more things that you can tweak, and it's nice to see something that will be living and keep going and knowing that it's got real, local experts." [Cycle 2; participant 1]<br>• "And that's nice touch. '...Real, genuine, expertise analysis.' This isn't some app that somebody who is going to sell me something...This screams, 'this is a legitimate researched, academically thought-out item.'" [Cycle 2; participant 1]<br>• "[Library]...I really like how deep you're going with the library and the beauty of that is that this can really become the place. And with having the scholarly article, with the simplified summary on top. Like, that's building like the library that, frankly, the ER doctors...the GP should go to because they're not going to take the time and need even the cardiologists...to read the amount of articles I've read in the last few months on ## syndrome. But maybe at least if you...could go something like this and go, 'Oh. OK, I read the summary. I've at least got the basic concept. Let's go.' So, I think you guys are on the right track." [Cycle 2; participant 1] |
| Relevant—applicability | • "[Library] The other piece I was going to say was useful...is the articles. So, you know, if I had a question, it would be nice to come to some content I knew is actually quite reliable, and, you know, |

| | |
|---|---|
| | might give me things in a way that was really accessible.” [Cycle 1; participant 4]<br>• “And I’ll tell you, if I had known about these symptoms ahead of time...I probably would have acted and not had to go through what I went through. But I didn’t make the connection. I didn’t make the connection of my heart palpitations are weird and that I can’t breathe properly when I’m going up a flight of stairs, and that I have really bad heartburn, and I’m sweating...If I'd made the connections, I would not be here today as far as in this situation where I had a heart attack and had a stroke. Right. So, I think it's good and if you've already had a cardiac event, it's even more important that you manage, if something starts happening again, that you know to pay attention.” [Cycle 2; participant 5]<br>• “[Chatbot] You’re asking the right questions. I really like the fact that it asks for the symptoms that you experienced and then it brings up those symptoms again and it makes that connection...It also brings up the things that are typically for women and not so much for men. I think it’s good that it’s specific for women because women are under researched and under diagnosed...If I had known that there were four or five things, that would have meant I had a heart problem, I would have paid closer attention...And I think the app’s good because it helps you put in the symptoms you’re feeling again and it’ll make you feel like you’re not being crazy, because sometimes you think, ‘should I go to ER or not go to the ER? What should I do?’ I feel like this and people are like, ‘Oh, it’s just nothing.’ But if I put it in my app, and I’ve put in four or five things that I’m feeling and it says, ‘oh, this is really close to what you experienced when you had your heart attack. You may want to really pay attention to this.’ That’s what I think is important.” [Cycle 2; participant 5] |

| | |
|---|---|
| | • "When you're panicking, and you're sitting in the hospital, and you're trying to remember all the things you need to tell...the person in the hospital—you could pull up your app and say, 'OK, so here's all the things,' right? Cause it helped you." [Cycle 2; participant 5]<br>• "[Chatbot] When you got all that heart pain, you need someone almost to guide you through the things that—or something you normally don't even think about, they're just natural abilities. So, it's kind of nice to tell someone—to tell [remind] me what to do [take nitroglycerin as prescribed]." [Cycle 2; participant 3] |
| Affirming—symptom recognition | • "[High-alert message] I think it's actually excellent. She doesn't know what to do, she doesn't want to go to the hospital. That's why someone is looking at an app—because you are wanting the app to tell you something different than what's happening and you want confirmation, so I think that this is good. The app says you need to call 911 so hopefully she does." [Cycle 1; participant 1]<br>• "[High-alert message] I thought that it made sense. It highlighted that you need to seek urgent care immediately based on what they had reported and then it tells you how to go about doing that and it reminds you why you are going to the ER...what's happening and why you're going. It covers all the bases." [Cycle 1; participant 2]<br>• "[High-alert message] I like that. Not for my situation, because I seem to—but I noticed that a lot of people don't know what to do. Then they go into panic and then they're—I don't know if you've seen that on the Facebook sites, so they start panicking and asking people, 'what I should do.'" [Cycle 2; participant 3]<br>• "[High-alert message] I'd probably be calling a family member or somebody. I would probably be following what you're saying, right? Calling 911. Yeah, that's a tough one because it's actually, it shows |

| | |
|---|---|
| | exactly what happened when I had my heart attack the first time and the only thing that made me call 911 was actually the fact that I couldn't breathe, which I didn't put down for this one, right. So...what you're saying is, this is close to what happened to you before, so you should really pay attention to it. So, I think it's a fair assessment." [Cycle 2; participant 5] |
| Personalized—social support | • "[Chatbot] But I think that actually the bots do quite a good job because we thought they were real until you guys told us that they weren't." [Cycle 1; participant 1]<br>• "[Chatbot] I just found it makes it more personal too. It feels like you are talking to a person and filling in fields and links and stuff." [Cycle 1; participant 2]<br>• "[Chatbot] It's sort of a really familiar way to kind of get asked a series of questions for someone who's used to sort of chatbots or texting or stuff like that. So having it be an interactive kind of thing is great." [Cycle 1; participant 4]<br>• "[Chatbot] I like the tone. It just feels accessible, it feels friendly, and when somebody is either want or wanting to learn something or is feeling scared, those kinds of thoughts, that's the tone that I would want." [Cycle 1; participant 4]<br>• "[Chatbot] I really love the bot feature. I think that's a real strength as it's immediately interactive, rather than, a lot of the times you can choose the bot or not. And I think with this, it's a really good way to go with it because it makes it really interactive and I think that's important, and it's gathering some data of the user." [Cycle 1; participant 5]<br>• "[Chatbot] And for some people, if they're really bad and they've got to go to the ER—some people are very, very alone. So even though this is a Holly robot, it's not a bad thing that Holly's going to |

| | |
|---|---|
| | reach out and say, ‘hey.’ An hour later, ‘How are you doing?’ because maybe Holly’s the only one who’s going to reach out and do that. And there’s still some comfort. It’s not, in some ways, that different from when I, at different times, I’d be actively messaging one of my heart groups online. If there was something so frustrating in the ER, I’ve got to share it with somebody and Holly could serve that purpose for some people. So, I think it’s—it wasn’t in pieces I expected, but I think it’s interesting.” [Cycle 2; participant 1]<br>• “[Videos]...I definitely enjoy, like Donna’s story here. I’m assuming that’s her life experience having the condition. I actually enjoy reading things like that. You help relate, it helps to—you’re not the only one, and then you relate to how someone else is feeling. And it, I like reading things like that. I think those are really good to have.” [Cycle 2; participant 3]<br>• “[Chatbot] But, when I’m in that state of mind and I’m dealing with the chest pains, I’m dealing with that, I actually like to be walked through things. It’s a calming. ‘Is that OK? Do this. Do this.’ And it actually helps. That is actually a really good way to do things.” [Cycle 2; participant 3]<br>• “[Videos and podcasts] No, I love the videos [personal testimonies]. Those are good.” [Cycle 2; participant 3] |
| Innovative—research and women’s heart health | • “I think there’s a lot of women on non-obstructive side, who would do this for sure. So, I think you’d get a lot of people saying, ‘well, yes, please study us, analyze us and figure us out.’” [Cycle 2; participant 1]<br>• “So, it becomes a place where I can do my own tracking, even if I don’t want to do a Heart Check or a Wellness check, I can come in here and make notes and keep it in a contained spot.” [Cycle 2; participant 1] |

| | |
|---|---|
| | • "I do like it and I think having women who are isolated...they don't have access to medical care that people in the GTA would have. I think it's excellent for that population because for them getting to see a doctor or getting to the hospital may not be as easy for them as it would for me. People who are living and have a limited income, taking the ambulance in Ontario costs $45, and that might be $45 someone doesn't want to spend. So having an app tell you need to go and you need to call 911 could be a deciding factor for them and it gets them going, as opposed to not doing anything, so I think that's a good target group." [Cycle 1; participant 1]<br>• "I found it fairly all-encompassing...It made me comfortable answering those questions and looking forward to the information it was going to provide, so that I could see if there is something in there to educate myself on the problems and why I was experiencing the symptoms I was experiencing, et cetera, and what I should do about it. I just found it comforting, you know, giving you access to education that not everybody would know where to find or start looking for. By being able to search even the meaning of a word in the context of an article, it would just make it much easier even when you went to speak to your physician to have something back you up rather than just hoping they understood what you were saying or hoping they read the same set or articles or have access to the same body of knowledge." [Cycle 1; participant 2]<br>• "Yeah, I would for sure still use it." [Cycle 1; participant 5] |
